# Patient-Reported Experiences in Vascular Surgery: A Qualitative Analysis of Care Quality

**DOI:** 10.1177/11786329251342283

**Published:** 2025-06-04

**Authors:** Maram Darwish, Katrin Abdelgafar, Sian Jackson, James Coulson, Kathleen Withers, David C. Bosanquet

**Affiliations:** 1 Southeast Wales Vascular Network, University Hospital of Wales, Cardiff, UK; 2 Health Education England, East Midlands Deanery, Nottingham, UK; 3 Morriston Hospital, Swansea Bay University Health Board, UK; 4 Vascular Surgery Department, Betsi Cadwaladr University Health Board, Bangor, UK; 5 Cardiff University, School of Medicine, University Hospital of Wales, Cardiff, UK; 6 CEDAR, Centre for Healthcare Evaluation, Device Assessment and Research, Cardiff and Vale University Health Board, UK; 7 School of Engineering, Cardiff University, UK; 8 The Welsh Value in Health Centre, Cwm Taf University Health Board, Mountain Ash, UK; 9 Department of Vascular Surgery, Aneurin Bevan University Health Board, Newport, UK

**Keywords:** vascular surgery, patient experience, patient reported experience measure, PREM, PREMs, qualitative research

## Abstract

**Background:**

The complexity of treatment pathways and the chronic nature of diseases in vascular surgery necessitate a patient-centred approach to improve care quality and health outcomes.

**Objectives:**

To explore vascular patients’ experiences, identifying key factors influencing their satisfaction and adherence to treatment.

**Study Design:**

Qualitative design using thematic analysis

**Methods:**

Seventeen semi-structured interviews were conducted with vascular patients (10 males and 7 females) from 3 vascular units in Wales. Proportional random sampling was used for participant selection based on recent vascular care, age, sex, and clinical backgrounds. Interviews were conducted in person, recorded, and transcribed verbatim. Thematic analysis was employed to identify key themes. NVivo 10 software facilitated data management.

**Results:**

Six themes were identified: (i) communication and information delivery, (ii) patient involvement in decision-making, (iii) pain management, (iv) psychological and emotional support, (v) healthcare environment and systemic processes and (vi) continuity of care and post-discharge experience. While patients generally appreciated the professionalism of the healthcare staff, notable disparities emerged in communication, particularly for patients with lower health literacy or during waiting periods. Inconsistencies in discharge planning and follow-up care highlighted systemic inequities. Across all units, patients reported a lack of formal psychological support. Involvement in decision-making varied, with some patients feeling adequately included, while others experienced exclusion and anxiety.

**Conclusion:**

These findings reveal key areas for improvement, with communication serving as a foundational element that enhances patient involvement in decision-making, psychological support, and continuity of care. Addressing these interconnected areas, with a focus on effective communication and health equity, may help close care gaps and improve outcomes for all vascular patients.

## Introduction

Understanding patient experience has increasingly become a vital aspect of effective healthcare delivery, encompassing the quality of interactions between patients and healthcare providers.^1-3^

In vascular surgery, the complexity of treatment pathways, the high-risk nature of procedures, and the chronicity of many vascular diseases necessitate a patient-centred approach.^4-7^ By capturing patients’ experiences, healthcare providers can identify strengths and areas for improvement, ultimately enhancing the quality of care.^7,8^ Prioritising patient experience leads to better clinical outcomes,^2,9,10^ as engaged and satisfied patients are more likely to adhere to treatment protocols and communicate openly with their providers.^8,10,11^

Additionally, understanding patient experiences in vascular surgery offers critical insights into disparities in care,^11^ particularly for vulnerable populations who may face barriers related to communication, access to services, and follow-up care.^6^ The complexity of vascular procedures often places an additional burden on patients from socioeconomically disadvantaged backgrounds, where health literacy, language barriers, or geographic distance from specialised care services can affect their experience.^6^

Existing Patient Reported Experience Measures (PREMs) developed for general surgery fail to adequately capture the complexities of vascular patient care. Unlike many surgical conditions where a single intervention resolves the issue, vascular diseases often require lifelong monitoring, staged interventions, and ongoing risk factor management. As such, a vascular-specific PREM is necessary to capture the longitudinal nature of care, the complexities of decision-making (including the interplay between conservative, endovascular, and open surgical options), and the importance of multidisciplinary management, factors that general surgical PREMs do not adequately address.^12^

Despite the growing recognition of the importance of patient experience in healthcare, there remains a lack of validated tools specifically designed to capture the unique experiences of vascular surgery patients.^12^ This study aims to explore these experiences, providing insights that can inform healthcare practices and enhance the patient journey in vascular care. While the findings will be used to contribute to the development of a vascular-specific PREM, the primary objective of this paper is to add to the knowledge base of patient experience in vascular surgery. This will identify key factors that can lead to meaningful improvements in care through informing clinical practices, healthcare policies, and educational strategies for healthcare professionals.

## Methodology

This qualitative study used semi-structured interviews to explore vascular surgery patients’ experiences as part of a broader effort to develop a vascular-specific PREM. Conducted across 3 major vascular units in Wales, each unit is managed under the All-Wales Vascular Network with many common pathways and management strategies, with only minor variations in care delivery (eg, vascular access service provision). These units serve distinct populations: South East Wales (served by Cardiff and Vale University Health Board [UHB], 1.56 million people people), South West Wales (served by Swansea Bay UHB, 1 million people), and North Wales (served by Betsi Cadwaladr UHB, 700 000 people), forming a network for independently managed patient care with complex cases very occasionally referred out of region when needed. Wales, with a total population of 3.1 million, is supported by this structure. Ethical approval (Reference: 24/PR/0522) was granted on 20/06/2024, with informed consent obtained from all participants. The study follows COREQ guidelines^13^ (Supplemental Document S1).

### Participants

Using proportional random sampling, 17 patients were selected from 3 vascular units across Wales, ensuring representation of diverse patient experiences. Selection was stratified to achieve a balance in age, gender, and clinical background. Specifically, we included a mix of emergency and elective admissions, as well as patients with different vascular conditions, including peripheral vascular disease, aortic disease, carotid pathology, and iatrogenic vascular injuries requiring surgical intervention. The sampling approach aimed to reflect real-world variations in patient experiences while ensuring coverage of key subgroups within vascular care. Inclusion criteria required adults (aged 18 or over) who had undergone a vascular procedure or inpatient care within the past 3 months, were cognitively able, provided informed consent, and could speak/read English or Welsh. All participants in this study, including those who underwent endovascular revascularization, were required to have a minimum of 24 hours of inpatient care. This criterion was set to ensure comprehensive capture of post-procedure experiences, including pain management, early complications, and transition to post-discharge care. Patients with iatrogenic vascular injuries were included if their definitive management was under the vascular surgery service, ensuring that the focus remained on their experience of vascular care rather than their primary surgical indication.

### Data Collection

Data collection took place from June to September 2024, shortly after each patient’s discharge. In-person interviews were prioritised to build rapport, with virtual and phone options available if preferred. Interviews lasted 30 to 60 minutes, guided by a structured tool (Supplemental Document S2) covering communication, decision-making, psychological support, and continuity of care. The guide was informed by a systematic review of surgical PREMs and qualitative studies.^12^ All interviews were conducted by MD, a female vascular surgery trainee with qualitative research experience, who had no prior relationships with participants to minimise bias. MD arranged each interview after initial contact by principal investigators (KA, SJ, DCB) and provided an overview of the study aims. All participants consented and completed the study.

Interviews were conducted at a location chosen for participant convenience, with all individuals opting for their treating hospital as the preferred setting. Each interview occurred in a private hospital room with only MD and the participant present, ensuring confidentiality. Each session concluded with MD taking field notes on non-verbal cues and observations to inform thematic analysis. Data collection continued until thematic saturation was achieved. Audio recordings, with participant consent, were transcribed in an intelligent verbatim format^14^ and anonymised to protect confidentiality.

### Data Analysis

#### Coding and Thematic Analysis Process

Thematic analysis was conducted following Braun and Clarke’s guidelines^15^ beginning with transcription and familiarisation through repeated transcript readings. Line-by-line coding combined inductive and deductive approaches: inductive coding allowed themes to emerge, while deductive coding linked findings to established concepts in vascular care PREMs.^15,16^

Initial codes were grouped into categories, forming preliminary themes through constant comparison across interviews. This iterative process involved continuous review and refinement to ensure themes accurately represented patient experiences, verified against raw data. NVivo 10 software^17^ facilitated code organisation, ensuring a systematic, traceable analysis. Thematic analysis allowed the identification of both overarching and condition-specific themes, ensuring that common experiences, as well as nuances related to different procedures, were adequately represented.^15^ Detailed coding information and representative transcript excerpts are available upon request.

### Qualitative Data Analysis

The 17 interviews followed an iterative thematic development process, progressing from simpler to more complex experiences until reaching thematic saturation. Early interviews provided foundational insights into patient satisfaction with communication, decision-making, and healthcare processes, introducing themes of patient autonomy and setting the stage for deeper exploration.

In line with established qualitative research methodologies, sample size was determined based on thematic saturation rather than statistical power calculations. Data collection continued until no new themes emerged, ensuring a comprehensive representation of patient experiences. The final sample size of 17 participants aligns with existing qualitative research standards, where saturation is typically reached within 12 to 20 interviews in focused healthcare studies.^15,18^

Themes were validated through member checking, with participants reviewing transcripts for accuracy. Discrepancies were incorporated, and research team discussions ensured consistency in theme development.

## Results

Table 1 depicts the demographic data of our study sample. Seventeen vascular patients were interviewed (10 males [59%] and 7 females [41%]), reflecting the male predominance in vascular disease (M: F 1.43:1). Ages ranged from 36 to 84 years, with a mean age of 65. Most participants had significant comorbidities: hypertension in 8 (47%), type 2 diabetes mellitus (T2DM) in 9 (53%), ischaemic heart disease (IHD) in 5 (29%), and chronic obstructive pulmonary disease (COPD) in 2 (12%). Thirteen (76%) were current or former smokers. Participants were treated for a range of vascular pathologies, including peripheral vascular disease (PVD) in 12, aortic disease in 2, carotid disease in 1, and iatrogenic vascular injuries in 2. Ten patients (59%) were admitted on an emergency basis, while 7 (41%) were admitted electively. Length of stay ranged from 2 to 88 days, with a mean of 15.5 days. The interval between discharge and interview ranged from 2 to 10 days, with a mean of 5.9 days, ensuring recency in patient reflections. Geographically, patients represented South East Wales (n = 9), South West Wales (n = 4), and North Wales (n = 4), receiving care across Wales’s 3 major vascular units.

**Table 1.** Participants demographic data.

Participant anonymised identifier	Age	Gender	Comorbidities	Smoking status	Admission type	Vascular pathology	Length of hospital stay	Time from discharge to interview	Geographical location
P1	72	M	IHD, HTN, T2DM	Ex smoker	Elective	PVD	9 d	4 d	South East Wales
P2	76	F	AF	Ex smoker	Emergency	PVD	13 d	7 d	South West Wales
P3	42	M	None	Nonsmoker	Emergency	PVD	8 d	2 d	South East Wales
P4	84	M	T2DM	Ex smoker	Elective	PVD	26 d	10 d	South West Wales
P5	62	M	IHD, T2DM, HTN	Ex smoker	Emergency	Iatrogenic injury	4 d	5 d	South East Wales
P6	53	M	HTN, lymphoma	Ex smoker	Emergency	PVD	88 d	8 d	South West Wales
P7	54	M	None	Ex smoker	Emergency	PVD	10 d	7 d	North Wales
P8	53	F	None	Ex smoker	Elective	PVD	3 d	4 d	South East Wales
P9	82	F	IHD, HTN, T2DM	Nonsmoker	Elective	PVD	33 d	9 d	South West Wales
P10	63	F	HTN, T2DM	Ex smoker	Emergency	Carotid disease	3 d	7 d	South East Wales
P11	36	M	T1DM	Smoker	Emergency	PVD	4 d	10 d	South East Wales
P12	71	M	HTN, HF, CLD, CKD, T2DM	Smoker	Elective	PVD	18 d	8 d	North Wales
P13	82	M	None	Ex smoker	Emergency	Aortic disease	16 d	3 d	North Wales
P14	61	M	T2DM, CREST	Ex smoker	Elective	PVD	6 d	8 d	North Wales
P15	76	F	T2DM, HTN, COPD	Smoker	Emergency	PVD	11 d	4 d	South East Wales
P16	72	F	IHD, T2DM, HTN, COPD	Ex smoker	Emergency	Iatrogenic injury	2 d	3 d	South West Wales
P17	68	F	HTN	Ex smoker	Elective	Aortic disease	10 d	2 d	South East Wales

Abbreviations: AF, atrial fibrillation; CKD, chronic kidney disease; CLD, chronic liver disease; COPD, chronic obstructive pulmonary disease; F, female; HF, heart failure; HTN, hypertension; IHD, ischaemic heart disease; M, male; PVD, peripheral vascular disease; T2DM, type 2 diabetes mellitus.

In preliminary interviews, patients reported significant communication breakdowns and conflicting medical advice, underscoring the psychological strain from inadequate information. Emotional distress was more pronounced among those with high-complexity cases, highlighting the importance of cohesive, timely communication for patient satisfaction and emotional well-being. Notably, patients with similar medical outcomes perceived care quality differently based on the transparency and frequency of communication. As interviews progressed, recurring themes of communication, continuity of care, and psychological support solidified. Later interviews reinforced findings on inadequate post-operative communication, long waiting times, and the emotional impacts of delayed or inconsistent care. Continuity of care emerged as pivotal to satisfaction, particularly for patients with long-term interactions within the healthcare system.

The final thematic analysis identified 6 core themes shaping the vascular patient experience: (1) Communication and Information Delivery, (2) Patient Involvement in Decision-Making, (3) Pain Management, (4) Psychological and Emotional Support, (5) Healthcare Environment and Systemic Processes, and (6) Continuity of Care and Post-Discharge Experience. Each theme is explored below with direct quotes to illustrate patients’ experiences.

### Communication and Information Delivery

The most pervasive theme across the interviews was the critical role of clear, timely, and consistent communication between patients and healthcare professionals. Participants provided numerous accounts illustrating the significance of clear communication, often contrasting positive experiences with those involving lapses. For instance, Participant P1 emphasised the thoroughness of explanations provided by his surgical team:

They explained everything like, ‘we will do this scan for this reason, then discuss it in a meeting.’ Right the way through, they kept me informed.

Such detailed communication appeared to be highly valued, as it allowed patients to better understand their treatment trajectory and manage their expectations.

Conversely, instances of insufficient communication were often cited as sources of distress.

P5 shared their frustration with receiving conflicting information from different doctors:

I was told by one doctor that I might lose my leg, and another came in later saying the chances were low. It was a relief, but why couldn’t they have told me this in the first place? It was like getting two completely different stories in a matter of days.

Similarly, P3 emphasised the lack of proactive communication during their hospital stay:

I sat there for four days waiting for an update. No one would tell me what was going on. It’s frustrating when you have to keep asking for information.

These sentiments underscore the importance of communication in reducing patient anxiety and enhancing trust in the medical team. During the study period, communication with patients about their treatment and care was primarily conducted through verbal consultations, supplemented by written patient information leaflets on specific vascular conditions and procedures. However, as our findings indicate, the consistency and delivery of this information varied across units.

### Patient Involvement in Decision-Making

Patient perspectives varied on involvement in treatment decisions. P7 felt pressured into surgery without options, stating:

I wasn’t given any real options. . . I wanted more time to understand.

In contrast, P13 and P17, who underwent elective surgery, appreciated the choice and time to ask questions, highlighting the importance of shared decision-making for all patients:

P13: They explained everything clearly and gave me time to ask questions. I knew what my options were, and that helped me feel more in control of my treatment.

P17: My doctor was brilliant; he didn’t rush me into anything. He laid everything in front of me and my husband, he gave me time to ask questions, and I felt like my concerns were heard. . . I felt confident and I trusted him.

This contrast highlights the varying degrees of patient involvement, with a clear need for providing adequate information and shared decision-making opportunities across all cases, whether elective or urgent.

### Pain Management

Pain management was a crucial aspect of patient well-being, affecting both physical and emotional health. Participants reported mixed experiences, with timely interventions often cited as critical. P7 expressed frustration with delayed postoperative pain control:

After my surgery, the pain was intense. I felt like I was begging. . . it was really hard to cope.

Similarly, P9 noted challenges with chronic pain, feeling misunderstood:

I deal with pain all the time. . . it’s exhausting, and sometimes I feel like the doctors just don’t understand.

Positive experiences were also shared, especially when healthcare providers were proactive. P17 found patient-controlled analgesia (PCA) empowering:

They started me on a button analgesia pump. . . it helped a lot.

PCA allowed patients control over their pain management, enhancing recovery. Additionally, P15 highlighted the importance of attentive staff:

The nurses checked in on my pain levels regularly. . . that made a big difference.

These accounts underscore the impact of patient-centred, responsive pain management strategies.

### Psychological and Emotional Support

Participants reported mixed experiences with emotional support. Some, like P8, appreciated providers’ empathy:

They were incredibly kind. . . it made all the difference.

Such encounters underscored the positive impact of emotional support on reducing anxiety. Others highlighted the emotional toll of unmanaged pain, emphasising a need for holistic approaches. P16 shared:

It’s not just physical pain; it weighs on your mind too. . . I wish there was more support.

The absence of mental health support during stressful periods was a common frustration. P2 and P3 expressed feeling isolated, with P2 stating:

No one asked if I wanted to speak to someone. . . it would have made a huge difference,

while P3 struggled with the emotional burden of uncommunicated risks:

I didn’t want to worry my wife, but I was scared. . . I couldn’t sleep for days.

These reflections indicate a potential need for integrating psychological support into patient care, especially in high-risk situations, alongside accessible, clear information. Notably, none of the participants in our study reported utilising such services, underscoring a potential gap in available psychological support for vascular patients.

### Healthcare Environmental and Systemic Processes

The physical environment, especially cleanliness, was frequently linked to feelings of safety. P9 appreciated this, noting:

Everything was spotless, which made me feel safe.

However, issues like noise and lack of privacy in shared wards detracted from patient comfort. P6 remarked:

It was hard to sleep with all the noise. . . I felt like I had no privacy.

These experiences point to areas for improvement in the hospital environment.

Systemic issues, such as staffing, scheduling, and waiting times, were common frustrations. Although some delays were unavoidable, patients felt that better communication could alleviate related stress. P3 shared:

I knew I’d have to wait, but I just wanted someone to tell me what was going on.

This highlights the role of communication in managing patient anxiety even when operational constraints persist.

Logistical efficiency also influenced satisfaction, with P17 valuing streamlined follow-ups:

They tried to schedule things so I wouldn’t have to keep coming back.

Similarly, continuity of care was affected by disjointed communication across departments. P12 noted:

Every time I saw a different doctor, I had to repeat my story. . . it felt like my care was just being passed around.

Such insights underscore the impact of both environmental and systemic factors on patient experience, with communication as a key factor in mitigating these stresses

### Continuity of Care and Post-Discharge Experience

While some vascular procedures, such as endovascular revascularization, are performed as day-case interventions in select centres, all patients in this study were admitted for at least 24 hours, as per national policy. This standard ensured that key aspects of early recovery, including access-site management, pain control, and observation for immediate complications, were consistent across participants.

Well-coordinated follow-up care was vital for patients’ recovery, providing reassurance and support. P13 commented:

Knowing I had follow-ups scheduled helped me feel more secure about my recovery.

This feeling of reassurance was common among those with clear post-operative guidance. However, some participants experienced challenges, particularly during transitions from hospital to home. Insufficient information on post-discharge care left some feeling unprepared and administrative issues complicated continuity for some, as P1 shared:

My medical records didn’t transfer properly. . . I had to commute back to England for my appointments.

Also, P10 described feeling rushed at discharge:

It would be beneficial to have a more structured follow-up. . . I had many questions.

These accounts underscore the importance of clear communication and proactive outreach during discharge, ensuring patients are well-equipped to manage their recovery and addressing any lingering concerns.

**Figure 1.**
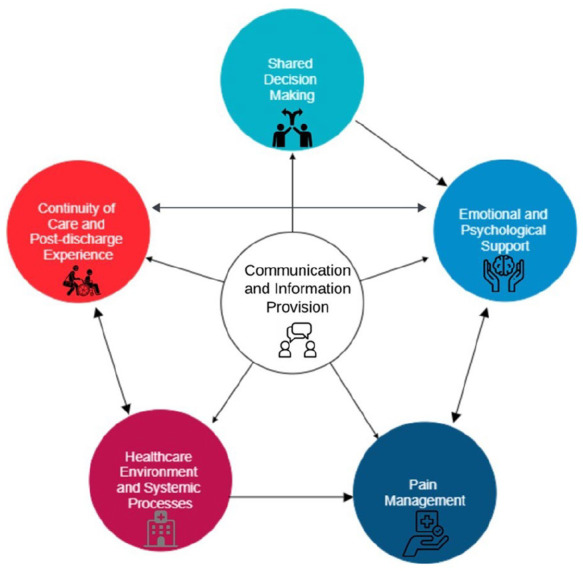
Interconnected themes of the vascular patient experience.

The findings highlighted communication, patient involvement in decision-making, psychological support, environmental and logistical support, and continuity of care as core components of the vascular patient experience. These themes are interconnected, with communication as the foundation influencing other areas. Effective communication facilitated decision-making, enhanced psychological support, and improved continuity of care, helping to reduce patient distress and foster trust in providers. Positive experiences arose when communication was clear and patients felt involved, while inconsistent information and limited support often led to negative experiences. These interrelations, with communication at its core, is illustrated in Figure 1

## Discussion

The study provides a detailed understanding of vascular surgery patient experiences, highlighting key factors that influence satisfaction. A key distinction in vascular care is its chronic, progressive nature, often necessitating long-term follow-up and repeat interventions. The need for ongoing monitoring and risk factor management applies not only to chronic conditions such as peripheral vascular disease but also to patients treated for aortic aneurysms, who typically require lifelong surveillance of graft integrity or aneurysm progression. Even in cases of iatrogenic vascular injuries, long-term follow-up is often necessary due to associated comorbidities and the need to prevent recurrence. However, it is important to note that follow-up patterns and workloads vary between conditions, and in some cases, surveillance is stopped after a short period depending on the stability of the condition and individual risk profiles. Nevertheless, the overarching requirement for engagement with vascular services, whether short-term or long-term, remains a shared characteristic across vascular patients, reinforcing the importance of experience-based monitoring frameworks.

While general surgical PREMs may assess perioperative satisfaction, they do not adequately capture the cyclical decision-making required in vascular disease management. Additionally, patients undergoing elective major procedures (eg, aortic repair) often enter surgery asymptomatic but with a high-risk pathology, whereas patients undergoing symptom-driven interventions (eg, PVD revascularization) typically experience substantial preoperative discomfort. These differences influence patient expectations, their perceptions of surgical benefit, and their recovery experiences. Our findings emphasise the need for a vascular-specific PREM that reflects these realities, ensuring that quality-of-care assessments align with the patient journey in this specialty.

Communication emerged as central, with patients expressing a desire for clear, timely information regarding diagnoses, treatment, and risks. Poor communication, especially during waiting periods, led to anxiety, and feelings of vulnerability during their hospital journey, underscoring the need for better communication protocols.

Shared decision-making was another area of importance. Patients who felt actively involved in their care decisions reported greater satisfaction and trust in their healthcare providers. Conversely, those who felt excluded felt frustrated and disempowered. This aligns with the principles of prudent healthcare and co-production^19^ and research showing the positive impact of shared decision-making on patient satisfaction and adherence to treatment plans.^20-24^

Pain management emerged as a critical determinant of patient experience, with most patients reporting positive experiences when pain was managed effectively. However, patients with complex pain histories reported dissatisfaction, highlighting the need for personalised pain management strategies. Also, the experiences shared by participants underscore the necessity for healthcare providers to prioritise effective pain management strategies, ensuring that patients receive timely interventions and support throughout their treatment journey. This finding aligns with existing research suggesting that effective pain management is a key factor in postoperative recovery and patient satisfaction.^25-27^ A comprehensive approach that includes both pharmacological and psychological support is essential for enhancing the overall patient experience in vascular care.

Systemic issues, such as understaffing and scheduling delays, were common sources of frustration for patients. Patients indicated that while waiting times were expected, proactive communication could help ease the anxiety associated with these delays. Implementing communication protocols to provide timely updates during unavoidable waits may bridge this gap, making patients feel more supported despite systemic constraints. Previous studies have shown that frequent updates and clear information can foster trust and reduce the emotional toll during prolonged waiting periods.^28-31^ Ensuring a comfortable, well-resourced environment, along with streamlined communication and care coordination, is essential for fostering a positive patient experience within vascular care.

The discharge process and follow-up care were similarly inconsistent, with some patients receiving clear instructions and others feeling inadequately prepared for self-management. Effective discharge planning, coupled with timely follow-up care, is essential for ensuring positive patient outcomes.^32-35^ The variation in experiences indicates a need for standardised discharge protocols that provide patients with clear, accessible instructions and ensure continuity of care post-discharge.

The emotional and psychological toll of vascular surgery was frequently noted. This suggests a gap in awareness or integration of available mental health services within the vascular care pathway, particularly for patients undergoing major surgeries. Enhancing visibility and accessibility of psychological support services could provide critical help for patients dealing with fear, anxiety, and the long-term impact of their condition.^36,37^

While the diversity of conditions within vascular surgery, ranging from aortic aneurysms to peripheral vascular disease and iatrogenic injuries, may suggest the need for condition-specific PREMs, our findings highlight a core set of shared experiential domains that transcend diagnosis. Communication, decision-making, continuity of care, and psychological support were prominent across patient narratives regardless of underlying pathology. This supports the feasibility of a unified vascular-specific PREM that captures these common experiences while allowing modular flexibility for future tailoring. We acknowledge, however, that the debate on whether a single PREM can adequately represent such a varied patient population remains open. Moreover, previous literature suggests that while specificity is valuable, overly fragmented PREMs may hinder clinical integration and reduce their utility in comparative outcome assessments.^38^ Further research may reveal condition-specific nuances that justify the development of tailored subscales within a unified framework.

## Strengths and Limitations

### Strengths

This study provides valuable insights into the experiences of vascular patients, an area with limited prior qualitative research, especially in the context of developing a PREM. Few studies have explored this aspect of vascular care, making our findings a crucial addition to the patient-centred care knowledge base within this specialty. Through in-depth interviews, we captured detailed patient perspectives that may guide improvements in vascular healthcare delivery. Consistency in data collection was maintained by having a single interviewer conduct all interviews, and conducting interviews shortly after discharge minimised recall bias, thereby enhancing data accuracy.

### Limitations

There are key limitations that should be acknowledged in the context of this qualitative study. While developing condition-specific PREMs for each vascular pathology (eg, peripheral vascular disease, aortic disease, carotid disease) may offer more targeted insights, such an approach is resource-intensive and may limit widespread implementation. Each condition-specific PREM would require separate research, psychometric validation, and rollout. Although our study identified common experiential themes across diagnoses, further research could explore whether modular or adaptable PREMs might better accommodate condition-specific nuances, including variations in preoperative symptom burden or urgency of presentation.

The inclusion of patients with iatrogenic vascular injuries may have introduced some variability, as their original surgical indication might influence perceptions of care. However, our analysis focused specifically on their experience under vascular teams, and their thematic responses were consistent with the broader cohort, particularly in domains such as communication, decision-making, and continuity of care. Further exploration of this subgroup could reveal whether distinct concerns warrant targeted evaluation. This study was conducted across vascular centres in Wales, which operate within a nationally coordinated network. While this structure provides consistency in care pathways, it may limit generalisability to other healthcare systems with different governance, funding, or service delivery models. Moreover, only English- and Welsh-speaking participants were included, potentially excluding perspectives from linguistically diverse populations.

Our sample included only inpatients with a minimum stay of 24 hours, thereby excluding day-case procedures, outpatient experiences, and patients managed conservatively. This criterion allowed in-depth exploration of perioperative care but may not reflect the full range of vascular experiences. Interviews were also limited to patients; input from family members or carers, who often play critical roles in decision-making and post-discharge support, was not captured.

Finally, data collection occurred between June and September 2024. Although vascular services operate continuously, we cannot exclude the potential influence of seasonal variations on hospital processes or patient experiences. These limitations highlight opportunities for further research to explore patient perspectives across outpatient settings, non-English-speaking populations, and international healthcare systems, and to determine the potential value of condition-specific or modular PREM frameworks.

## Conclusion and Clinical Implications

The findings of this study carry significant implications for clinicians involved in vascular patients’ care. First, ensuring that patients are well-informed and feel involved in decision-making can enhance trust and improve patient satisfaction.^39^ For vascular surgeons, this means allocating sufficient time during consultations to address patient concerns and ensuring that written materials are provided to reinforce verbal explanations.

The variability in pain management experiences emphasises the need for personalised pain management strategies that account for individual patient factors. Surgeons should work closely with pain management teams to ensure that patients, particularly those with complex conditions, receive adequate and tailored pain relief both during their hospital stay and after discharge.

The study also underscores the impact of systemic issues, such as understaffing and extended waiting times, which can directly impact patient experience and outcomes. While national standards, such as targets for Critical Limb-Threatening Ischaemia (CLTI)^40^ and tr Carotid Endarterectomy (CEA),^41^ set clear timelines for key vascular procedures, achieving these standards consistently requires adequate resources and staffing. Vascular surgeons, as key stakeholders in the care process, can advocate for resource allocation that supports these targets, ensuring patients receive timely, high-quality care. Additionally, the Provision of Vascular Services (POVS) document^42^ highlights the value of integrating psychological support within the vascular care pathway, particularly for patients undergoing high-risk procedures. However, in our study, none of the participants reported accessing these services, suggesting a potential gap in awareness or accessibility. Vascular surgeons, as key stakeholders in the care process, can advocate for resource allocation to meet these procedural and psychological care standards, ensuring a more holistic approach that addresses both the physical and emotional needs of vascular patients.

Finally, this study highlights inconsistencies in the care experiences of vascular patients, with some receiving excellent care while others faced significant challenges, despite being treated within the same healthcare system. These differences suggest a need to ensure that all patients, regardless of their specific circumstances or location, receive consistent, high-quality care. By using these insights to guide future healthcare delivery models, providers can better address variations in care and support underserved patient populations, ensuring equitable access to the highest standard of care.

## Supplemental Material

sj-docx-1-his-10.1177_11786329251342283 – Supplemental material for Patient-Reported Experiences in Vascular Surgery: A Qualitative Analysis of Care Quality Supplemental material, sj-docx-1-his-10.1177_11786329251342283 for Patient-Reported Experiences in Vascular Surgery: A Qualitative Analysis of Care Quality by Maram Darwish, Katrin Abdelgafar, Sian Jackson, James Coulson, Kathleen Withers and David C. Bosanquet in Health Services Insights

sj-docx-2-his-10.1177_11786329251342283 – Supplemental material for Patient-Reported Experiences in Vascular Surgery: A Qualitative Analysis of Care Quality Supplemental material, sj-docx-2-his-10.1177_11786329251342283 for Patient-Reported Experiences in Vascular Surgery: A Qualitative Analysis of Care Quality by Maram Darwish, Katrin Abdelgafar, Sian Jackson, James Coulson, Kathleen Withers and David C. Bosanquet in Health Services Insights
