# Supplementary material for: Patient-Reported Experiences in Vascular Surgery: A Qualitative Analysis of Care Quality
Source: Health Serv Insights. 2025 Jun 4;18:11786329251342283. doi: 10.1177/11786329251342283 (PMC12138208; doi:10.1177/11786329251342283)
Supplement: sj-docx-1-his-10.1177_11786329251342283 – Supplemental material for Patient-Reported Experiences in Vascular Surgery: A Qualitative Analysis of Care Quality [file sj-docx-1-his-10.1177_11786329251342283.docx]

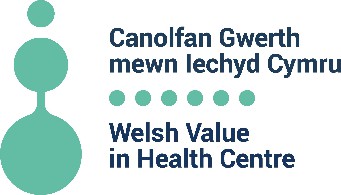

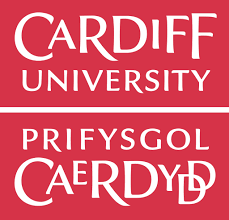


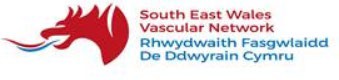

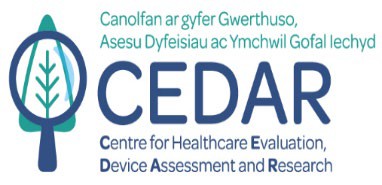

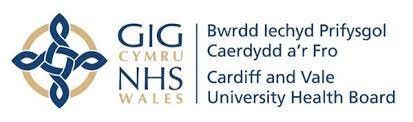


Patients Interview Guide

***Welcome and instructions.***

Thank you for agreeing to participate in this interview to discuss your experience in vascular surgery. The purpose is to understand your journey with the vascular surgery team and in the vascular unit. Prior to this session, you were provided with a participant information sheet and consented over the phone. Here's what to expect:

1. Your identity will remain conﬁdential.
2. I will ask you questions about your vascular surgery journey from diagnosis onwards to initiate our discussion.
3. I will facilitate the interview, track time, and ensure key topics are covered.
4. I will not actively contribute or interrupt you but will provide clariﬁcations when needed.
5. The conversation will be recorded for accuracy. Please speak clearly as the recorder won't capture nods or gestures.
6. Before delving into the topic, I and any member of the team will introduce ourselves and conﬁrm the recording setup.
7. Rest assured; the discussion will be anonymous. Recordings will be securely stored on a password-protected NHS server, transcribed verbatim, and destroyed at the end of the study. The transcribed notes will not link you to speciﬁc statements.
8. Please share your experiences truthfully.

# Ground Rules:

1. There are no right or wrong answers.
2. Share your thoughts as they come; no speciﬁc order required.
3. Your views are important; please participate and share your thoughts in as much details as you wish.
4. You are not obligated to answer any question if you don’t feel comfortable to.

# Any questions before we begin?

**Consent**:

Are you still willing to consent to participating in this study?

# Introduction:

Please introduce yourself, share your vascular disease, and how long you have been visiting the vascular surgery service.

# Playback:

We will playback the recording to ensure all voices are recorded clearly.

…

1. Introduction
   - Can you share your understanding of the purpose of this interview?
   - How would you describe the importance of the patient reported experience measure (PREM)?
2. Demographics
   - What is your age, gender, occupation, and any other relevant demographic details you would like to share?
3. Medical History
   - Can you provide a brief overview of your vascular condition and any history of vascular surgery?
4. Pre-Surgery Experience
   - What were the initial symptoms or signs that led to the necessity of vascular surgery for you?
   - Could you describe the diagnostic process and your conversations with healthcare professionals leading up to the surgery?
5. Decision-Making Process
   - What factors did you consider when deciding to undergo vascular surgery?
   - How would you describe the decision-making process, including your discussions with family, friends, and healthcare providers?
6. Surgical Journey
   - Can you provide a description of the surgical procedure and your experience during the recovery process?
   - How would you describe your hospital experience, including interactions with the medical staff and the overall environment?
7. Post-Surgery Experience
   - What challenges and adjustments did you face after the surgery?
   - How was the rehabilitation process, and did you experience any lifestyle changes post- surgery?
8. Support System
   - How did your family, friends, and healthcare professionals support you during your surgical journey?
9. Quality of Life
   - How has the vascular surgery impacted your quality of life?
   - Could you share how it affected your return to daily activities and any limitations you experienced?
10. Satisfaction with Care
    - What feedback do you have on the overall care you received, including communication, information provision, and coordination of care?
11. Future Expectations
    - What are your hopes, concerns, and expectations for the future regarding your vascular health?
12. Closing
    - Is there any additional comment or feedback you would like to share?
    - We want to thank you for your participation and valuable input.
